# Nuclear mTOR Signaling Orchestrates Transcriptional Programs Underlying Cellular Growth and Metabolism

**DOI:** 10.3390/cells13090781

**Published:** 2024-05-03

**Authors:** Tinghan Zhao, Jialin Fan, Ahmed Abu-Zaid, Stephen K. Burley, X.F. Steven Zheng

**Affiliations:** 1 Rutgers Cancer Institute of New Jersey, Rutgers, The State University of New Jersey, New Brunswick, NJ 08901, USA; 2 RCSB Protein Data Bank and Institute for Quantitative Biomedicine, Rutgers, The State University of New Jersey, 174 Frelinghuysen Road, Piscataway, NJ 08854, USA; 3 Department of Chemistry and Chemical Biology, Rutgers, The State University of New Jersey, 174 Frelinghuysen Road, Piscataway, NJ 08854, USA; 4 Department of Pharmacology, Robert Wood Johnson Medical School, Rutgers, The State University of New Jersey, Piscataway, NJ 08854, USA

**Keywords:** mTOR, signaling, rapamycin, androgen receptor, ARID1A, cBAF, nucleus, transcription, epigenetics, chromatin, growth, metabolism, histone methylation, histone acetylation, chromatin remodeling, cancer

## Abstract

mTOR is a central regulator of cell growth and metabolism in response to mitogenic and nutrient signals. Notably, mTOR is not only found in the cytoplasm but also in the nucleus. This review highlights direct involvement of nuclear mTOR in regulating transcription factors, orchestrating epigenetic modifications, and facilitating chromatin remodeling. These effects intricately modulate gene expression programs associated with growth and metabolic processes. Furthermore, the review underscores the importance of nuclear mTOR in mediating the interplay between metabolism and epigenetic modifications. By integrating its functions in nutrient signaling and gene expression related to growth and metabolism, nuclear mTOR emerges as a central hub governing cellular homeostasis, malignant transformation, and cancer progression. Better understanding of nuclear mTOR signaling has the potential to lead to novel therapies against cancer and other growth-related diseases.

## 1. Overview of mTOR

Mechanistic target of rapamycin (mTOR) is a highly conserved protein serine/threonine kinase. It is a member of the phosphatidylinositol-3-kinase-related kinase (PIKK) family. mTOR plays a pivotal role in regulating cell growth and metabolism across eukaryotic organisms [1,2,3]. mTOR is the core component of two distinct protein complexes, mTOR complex 1 (mTORC1) and mTOR complex 2 (mTORC2) [1,2,3]. mTORC1 senses signals from nutrients, growth factors, and hormones, controlling cellular growth and metabolism [1,2]. On the other hand, mTORC2 primarily responds to growth factors, thereby regulating cell proliferation and survival [1,2]. mTORC2 also serves as a conserved regulator of mechano-signaling homeostasis throughout evolution. Stretch tensions stimulate mTORC2-dependent responses, leading to an increase in plasma membrane surface area, response to shear stress, and changes in cytoskeletal programs, facilitating efficient coordination of cellular shape and movement [4,5,6].

Dysregulation of the mTOR pathway is implicated in a spectrum of pathophysiological conditions, ranging from premature aging and neurodegenerative diseases like Alzheimer’s to metabolic disorders such as diabetes and obesity, as well as cancer [1,2,3]. This intricate involvement of mTOR dysregulation underscores its broad impact on human health. Aberrant activation of mTOR signaling pathways can disrupt normal cellular metabolic homeostasis, leading to accelerated aging processes, metabolic disorders like diabetes and obesity, and notably, the development and progression of cancer. It is estimated that hyperactivation of mTOR signaling occurs in more than 50% of human tumors, promoting uncontrolled cell growth, metabolism, and survival [1,2,3].

**Figure 1:**
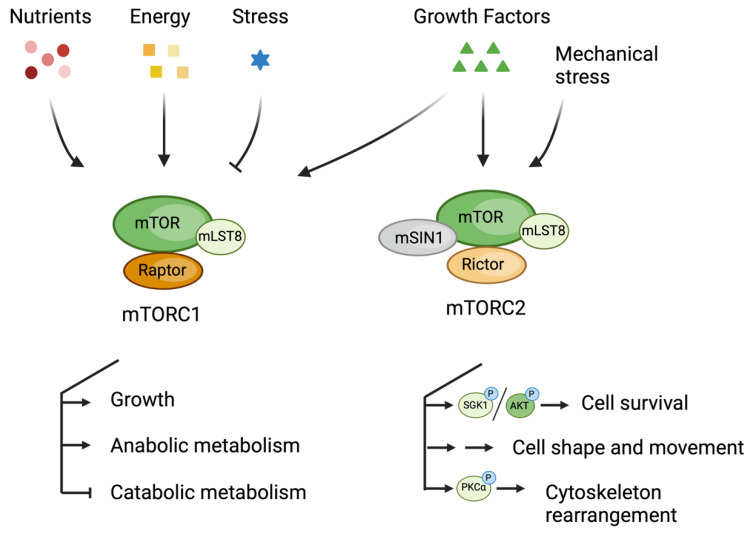
Schematic model showing mTORC1 and mTORC2, their main upstream signals, and downstream cellular events.

mTORC1 comprises three core components: mTOR, Raptor, and mLST8 (also known as GβL) (Figure 1) [1,2]. Raptor is crucial for mTORC1 assembly and substrate recruitment. It is exclusive to mTORC1. The interaction between Raptor and mTOR plays a regulatory role in nutrient-stimulated signaling, enhancing mTORC1 kinase activity and substrate specificity by binding to the TOR signaling motif (TOS). In contrast, mLST8 is a shared component of both mTORC1 and mTORC2. mTORC2 consists of mTOR and mLST8, but instead of Raptor, it includes two distinct core components: RICTOR and mSIN1 (Figure 1) [1,2,3]. RICTOR is essential for the assembly, substrate recognition, and stability of mTORC2. mSIN1 is an integral component of mTORC2 necessary for the assembly of the complex.

mTOR, as a crucial nexus governing cellular growth, metabolism, and proliferation, is an established drug target, particularly in cancer. At the forefront of mTOR-targeted therapy lies rapamycin and its analogs (rapalogs) [7,8]. Rapamycin is a macrolide antibiotic that was originally developed as an immunosuppressant. It serves as the prototypical mTOR inhibitor. Its mechanism of action (moa) involves binding to the immunophilin FKBP12, forming a complex that allosterically inhibits mTORC1. It was also reported that rapamycin could indirectly interfere with mTORC2 activity under certain situations [9,10]. Rapalogs, including temsirolimus and everolimus, are semi-synthetic analogs of rapamycin. They utilize the same moa but possess improved pharmacokinetic properties. By selectively targeting mTORC1, rapalogs impede aberrant cell growth and proliferation, thereby exhibiting anticancer effects. Beyond oncology, rapalogs are clinically used as immunosupressants for organ transplantation, treatment of Tuberous Sclerosis Complex (TSC)-associated disorders, and in drug-eluting stents to prevent restenosis. They also hold therapeutic potential in a broad range of other diseases characterized by mTOR dysregulation, including neurological conditions.

## 2. Nuclear mTOR Signaling

mTOR is found in diverse subcellular localizations, including the endoplasmic reticulum (ER), the Golgi apparatus, lysosomes, mitochondria, and the nucleus [11,12]. mTOR has been predominantly perceived as a cytoplasmic signaling molecule, owing to early focus on its role in mRNA translation and prevalent use of HEK293 cells as a model system, wherein mTOR’s nuclear localization is relatively low [11]. More recently, however, accumulating evidence has shed light on nuclear localization of mTOR and mTORC1 components across a broad spectrum of organisms and various cell and tissue types [13,14,15,16,17,18,19,20]. The presence of mTOR in both cytoplasm and nuclei offers new avenues for exploring the full scope of mTOR’s roles in cellular physiology and pathology (for recent reviews, see [11,21,22]).

Due to the prominent nuclear localization of mTOR, considerable efforts have been dedicated to characterizing possible interactions between nuclear mTOR and the chromatin. In yeast, nuclear localization of TOR has been shown to play a crucial role in rRNA expression. Using chromatin immunoprecipitation (ChIP), it was demonstrated that TOR directly associates with the promoter of ribosomal RNA (rRNA) genes in response to both starvation and rapamycin [16]. Importantly, chromatin-binding is critical for TOR to regulate rRNA transcription. This study provided the initial evidence of a functional role of TORC1 within the nucleus. Similar investigations have revealed mTOR binding to a diverse array of Pol I and Pol III transcribed genes, including those coding for 5S and 45S ribosomal RNAs (rRNAs), transfer RNAs (tRNAs), and U6 small nuclear RNA (snRNA) [23,24]. mTOR occupancy on the promoter of Pol III-transcribed genes is highly sensitive to rapamycin treatment across various cell lines (e.g., HEK293, HeLa, and C2C12), indicating a regulatory role of mTOR chromatin binding during transcriptional modulation [23,24].

Subsequent studies have demonstrated that mTORC1 also binds to diverse gene promoters transcribed by Pol II. For example, ChIP analysis revealed that both mTOR and Raptor associate with the promoters of PGC-1α and certain mitochondrial genes, modulating their expression through the transcription factor YY1 [25]. Additionally, mTOR was found to bind to the dystrophin promoter, enhancing dystrophin expression [26]. Muscle-specific inactivation of mTOR results in diminished muscle dystrophin content and severe myopathy [26]. Moreover, mTORC1 binds to the promoter of the long non-coding RNA (lncRNA) NEAT1, regulating NEAT1 transcription crucial for the biogenesis of nuclear paraspeckles [27]. Notably, ChIP-sequencing studies revealed that mTOR directly engages with thousands of regulatory regions of Pol II-transcribed genes in both mouse liver and human prostate cancer cells [28,29,30], suggesting profound regulatory roles of mTOR across a plethora of transcriptional programs.

As discussed in subsequent sections, mTORC1’s canonical role as a protein kinase is intricately involved in transcriptional regulation. For instance, mTORC1 binds to and phosphorylates key transcription factors such as the androgen receptor (AR) [31] and the estrogen receptor (ER) [32], modulating their transcriptional activities. Beyond its catalytic functions, mTORC1 also exhibits non-catalytic roles in gene expression. An illustrative example is its regulation of dystrophin transcription, which occurs in a cell-autonomous manner and is resistant to rapamycin, indicating a kinase-independent mechanism [26]. This suggests that mTORC1 possesses an intrinsic transcriptional function that extends beyond its classical kinase activity. Therefore, mTORC1 not only acts as a kinase, but also plays a nuanced role in directly influencing gene expression, contributing significantly to cellular regulatory networks.

Significant strides have been made in understanding how mitogenic and nutrient signals regulate cytoplasmic mTORC1. However, the mechanisms governing nuclear mTORC1 remain less clear. Recent studies have begun to shed light on this by using biosensors designed to specifically monitor mTORC1 kinase activity within the nucleus. These biosensors utilize mTORC1 substrate-phosphorylation-dependent fluorescence, allowing for the investigation of nuclear mTORC1 signaling dynamics in live cells [33,34]. Findings from these studies reveal that both growth factors and amino acids can rapidly induce nuclear mTORC1 substrate phosphorylation within minutes, showing kinetics comparable to cytosolic mTORC1 activation [33,34]. This research confirms that nuclear mTORC1 functions as an active protein kinase, aligning with earlier observations of kinase activity by mTORC1 isolated from the nuclear fraction towards 4E-BP1 in vitro [20]. These results strongly support the functional presence of mTORC1 kinase activity in the nucleus.

In yeast, Tor1 quickly relocates from the cytoplasm to the nucleus upon nutrient stimulation, which is blocked by rapamycin. This finding offers a clear mechanism for nutrient-driven Torc1 signaling into the nucleus [16]. After serum-deprivation-induced synchronization in IMR-90 lung fibroblasts, it was observed that nuclear mTOR became enriched upon release from cell cycle arrest, as evidenced by subcellular fractionation [35]. This discovery implies that mitogenic stimuli can also regulate mTOR’s nuclear localization. To thoroughly explore the localization of various components within mTOR complexes and their upstream regulators under physiological conditions, more systematic methodologies are required. Experiments using AKT, which localizes differently within cells, showed that nuclear mTORC1 activity triggered by growth factors depends on nuclear AKT activity [36]. This process facilitates the nuclear translocation of Raptor, a crucial component of mTORC1. Interestingly, Raptor’s nuclear localization seems adequate to sustain mTORC1 activity within the nucleus, even without growth factor stimulation.

Several studies have explored additional regulators of mTORC1. The small GTPase RHEB, which is essential for cytoplasmic mTORC1 activity, has also been observed in the nucleus. This nuclear localization plays a crucial role in nuclear mTORC1 activity independently of farnesylation [37]. Furthermore, nuclear RHEB is under the regulation of TSC2 [37], an upstream negative regulator of both RHEB and mTORC1. Additionally, two other mTORC1 regulators, Deptor and PRAS40, have been found in the nucleus, indicating their involvement in regulating nuclear mTORC1 signaling as well [38,39,40]. These findings indicate that mTORC1 is largely regulated by the same set of upstream signaling molecules. It remains to be established how the signal transduction cascade is spatially organized into the nucleus. Furthermore, it is crucial to determine whether these insights from engineered systems reflect common regulatory mechanisms across various cell types and tissues within their native cellular environments.

## 3. mTOR Governs Transcriptional Control of Cellular Growth Programs

mTORC1 integrates signals from growth factors, nutrients, and oncogenic signals to promote cellular growth. Ribosomes are the cellular machinery responsible for synthesizing proteins, which play an essential role in supporting cellular growth [41]. In particular, rapidly growing cancer cells require a large number of ribosomes to support the high demand for protein synthesis [42]. Ribosome biogenesis is the process by which ribosomes are produced. It involves transcription of ribosomal RNAs (rRNAs) by Pol I and Pol III, and ribosomal proteins by Pol II, processing of pre-rRNA into mature rRNA, and assembly of ribosomal subunits [42]. It is estimated that ribosome biogenesis accounts for up to 90% of nuclear transcription, which is a high-energy-consuming event that requires tight regulation in order to adapt to changing environmental conditions and cellular needs. mTORC1 regulates Pol I transcription of the large rRNA precursor that is subsequently processed into mature 28S, 18S, and 5.8S rRNAs [16,23,43,44,45,46], and Pol III transcription of the 5S rRNA [23,24,46,47,48,49,50,51,52,53]. These rRNAs are core components of the ribosome. Additionally, mTORC1 controls transcription of transfer RNAs (tRNAs) by Pol III [23,24,46,47,48,49,50,51,52,53], which also plays an essential role in facilitating mRNA translation into polypeptides.

**Figure 2:**
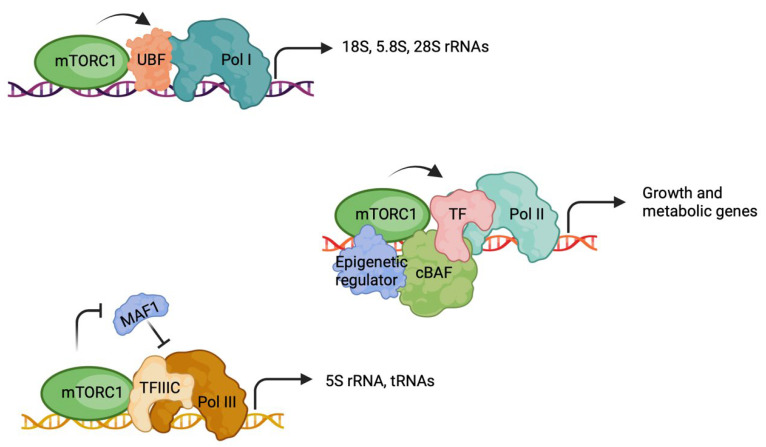
Nuclear mTORC1 regulates transcription by all three major RNA polymerases through diverse mechanisms. TF, transcription factor.

mTORC1 regulates Pol I activity through S6K1-mediated phosphorylation of the transcription factor UBF (upstream binding factor) (Figure 2). Subsequently, UBF binds to the rDNA promoter, recruiting Pol I and initiating transcription of rRNA genes [54]. Moreover, TORC1 signaling modulates condensin-mediated rDNA chromatin condensation, impacting the stability of rDNA tandem arrays and Pol I transcription [43,45,55]. mTORC1 also regulates MAF1, a repressor of Pol III-dependent transcription involving tRNAs, 5S rRNA, and certain small nuclear RNAs [23,24,47,48,49,50,51,52] (Figure 2). mTORC1 directly phosphorylates MAF1, inhibiting its repressor function on Pol III activity [24,49,50,51,52]. This post-translation modification (PTM) also induces MAF1 sequestration in the cytoplasm, further promoting Pol III transcription [56]. Additionally, mTOR interacts with TFIIIC, a DNA-binding protein that recognizes the promoters of these genes. TFIIIC contains a TOS that facilitates association with mTOR [24].

mTOR plays a crucial role in phosphorylating various transcription factors associated with Pol II, regulating cellular growth and metabolism (Figure 2). For example, mTORC1 interacts with and phosphorylates both the androgen receptor (AR) and the estrogen receptor (ER), the male and female hormone receptor transcription factors, respectively. In hepatocytes, mTORC1 interacts with AR and phosphorylates AR at S96 [31]. This interaction and regulation enhance AR stability, nuclear localization, and transcriptional activity towards multiple growth pathways, such as IGF1, PI3K-AKT, and β-Catenin, thereby promoting hepatocyte growth, proliferation, and liver tumorigenesis [31,57,58]. Similarly, mTORC1 interacts with AR in prostate cancer cells and androgens reprogram mTOR–chromatin associations in an AR-dependent manner [28,30,59,60]. mTOR also directly phosphorylates S104/106 of ERα in breast cancer cell lines, resulting in its activation and the upregulation of estrogenic gene transcription, thereby promoting growth and proliferation [32].

Another instance is mTORC1 phosphorylation of STAT3 at S727. STAT3 is a transcription factor crucial for embryonic development, tissue growth, and survival [61]. S727 phosphorylation yields maximal activation of STAT3 by CNTF in neuroblastoma cells. mTOR also phosphorylates TFEB, resulting in its cytoplasmic retention and reduced expression of TFEB target genes [62], which are involved in lysosomal biogenesis, autophagosome formation, and endocytosis. The mTORC1–MAF1 axis has recently been shown to regulate genes transcribed by Pol II, including PTEN in hepatocytes [63], and CREB-associated genes in the central nervous system (CNS) and retina neurons [47,64]. These genes are critical for promoting hepatocyte growth and CNS plasticity during neurodevelopment and neural repair. Interestingly, MAF1 can serve as both a transcriptional activator and a repressor of Pol II, depending on specific target genes.

## 4. mTOR Governs Transcriptional Control of Cellular Metabolism

In support of cellular growth, mTORC1 facilitates anabolic processes such as transport of nutrients and synthesis of amino acids, lipids, and nucleotides [1,2]. Meanwhile, it inhibits catabolic activities by suppressing autophagy and lysosome-mediated degradation [1,2]. Conversely, nutrient deprivation dampens mTORC1 activity, leading to decreased anabolic processes to conserve energy and resources. Additionally, mTORC1 inhibition upregulates autophagy, which recycles redundant cellular components (e.g., ribosomes and mitochondria) to liberate nutrients necessary for sustaining survival [1,2]. As described below, mTORC1 plays a major role in programming expression of genes related to cellular metabolism by regulating specific transcription factors and target genes.

Glucose is a crucial nutrient that supports cellular metabolism. Glucose enhances flux through glycolysis, serves as a primary source of energy for cells, and generates many intermediates for biosynthetic pathways. Malignant cells exhibit enhanced glycolysis in the presence of oxygen. This phenomenon is called aerobic glycolysis or the Warburg effect [65,66]. mTORC1 stimulates glucose uptake and anerobic glycolysis in cancer cells by regulating hypoxia-inducible factor (HIF1α) and MYC [67,68,69,70,71]. These structurally similar basic helix–loop–helix transcription factors, in turn, enhance expression of glucose transporters (GLUT), Hexokinase 2 (HK2), pyruvate kinase M2 (PKM2), and lactate dehydrogenase (LDH), which are key glucose transport and utilization enzymes. mTORC1 also mediates transcriptional repression of the long non-coding RNA (lncRNA) NEAT1 and NEAT1-mediated nuclear paraspeckle biogenesis [27]. Nuclear paraspeckles inhibit aerobic glycolysis via sequestration of NONO, which is needed for maturation of GLUT1, HK2, and LDH pre-mRNAs [27]. Upon oncogenic activation of mTORC1, NONO is released from nuclear paraspeckles, increasing expression of these key glucose transport and utilization enzymes and promoting aerobic glycolysis to support hyperactive cellular metabolism characteristic of cancer cells [27].

Lipids are important for the formation of cellular membranes and organelles. They also serve as signaling molecules and a means of energy storage. mTORC1 is a key regulator of lipid synthesis through modulation of SREBP1, which is a basic helix–loop–helix, leucine zipper transcription factor that controls the expression of genes related to cholesterol and fatty acid biosynthesis. mTORC1 regulates SREBP1 both transcriptionally and post-translationally. In hepatocytes, insulin stimulates expression of SREBP1 in an mTORC1-dependent manner [72]. Interestingly, four androgen response element (ARE) sequence motifs occur in the SREPB1 promoter. AR phosphorylation by mTORC1 at S96 stimulates SREBP1 transcription and expression of SREBP1-dependent lipogenic genes [31]. Under conditions of low sterol levels, SREBP1 becomes activated. The mTORC1 signaling cascade stimulates lipid synthesis in two distinct mechanisms. mTORC1 activates SREBP1 through S6K1-mediated phosphorylation, leading to translocation of active SREBP into the nucleus for gene upregulation. Another relevant PTM involves mTORC1 phosphorylation and inhibition of Lipin1, a negative regulator of SREBP1.

mTORC1 promotes nucleotide biosynthesis, particularly in proliferative cells, to support DNA replication and ribosome biogenesis. mTORC1 upregulates expression of methylenetetrahydrofolate dehydrogenase 2 (MTHFD2) through the basic leucine-zipper transcription factor ATF-4 [73]. MTHFD2 is an important enzyme in mitochondrial folate one-carbon metabolism, providing carbon units necessary for purine synthesis.

mTORC1 plays a key role in regulating amino acid transport and metabolism. The mTOR–ATF4 axis also promotes serine/one-carbon metabolism [74], which supplies important metabolites, including NADPH and S-adenosylmethionine (SAM). mTOR phosphorylates TFEB, causing cytoplasmic retention of TFEB and reduced expression of TFEB target genes, including those involved in lysosomal biogenesis, autophagosome formation, and endocytosis [62]. Conversely, repression of mTORC1 by starvation triggers activation of TFEB, which enhances lysosomal biogenesis and autophagy. Activation of autophagy leads to lysosomal degradation of ribosomes, recycling them into free amino acids and ribonucleotides. Glutamine, an abundant non-essential amino acid, plays a crucial role in replenishing tricarboxylic acid (TCA) cycle intermediates via glutaminolysis. mTORC1 stimulates glutamine metabolism by modulating MYC-dependent transcription of genes related to glutaminolysis, including glutaminase 2 [75]. Additionally, mTORC1 promotes glutaminolysis by facilitating CREB2-regulated transcription of SIRT4 [76].

Amino acids have recently been demonstrated to activate mTORC1 via a RAB1A-dependent mechanism [77]. Intriguingly, branched-chain amino acids (BCAAs), acting through the RAB1A–mTORC1 complex, enhance the stability and nuclear localization of PDX1, a key transcription factor governing the growth, function, and identity of pancreatic β-cells [78,79]. Activation of PDX1 by mTORC1 enhances insulin transcription and circulating insulin. This discovery unveils a novel role and the underlying mechanism through which amino acids regulate the body’s glucose levels and insulin-stimulated tissue metabolism via a beta-cell-specific function mediated by the RAB1A–mTORC1–PDX1 signaling axis.

## 5. mTOR Controls Epigenetic Modifications and Chromatin Remodeling

**Figure 3:**
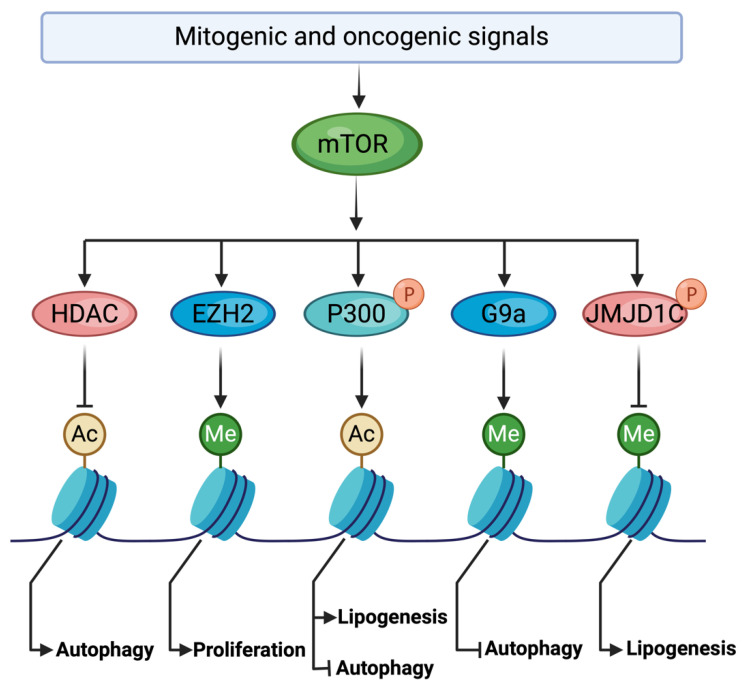
mTOR regulates growth and metabolic epigenome through diverse mechanisms. P, phosphorylation; Me, methylation; Ac, acetylation.

Histone modifications play a pivotal role in dynamically regulating gene expression [80,81]. Methylation of histone lysine and arginine residues is orchestrated by histone methyltransferases (HMTs) and histone demethylases (KDMs) [82]. Emerging research has identified mTOR as a direct regulator of the intricate network governing epigenetic modifications and chromatin dynamics (Figure 3). Enhancer of Zester 2 (EZH2), a catalytic component of the Polycomb repressive complex 2 (PRC2), is responsible for the di- and tri-methylation of H3K27 (H3K27me2 and H3K27me3) via its SET-domain-containing lysine HMT [83]. EZH2, the target of the anti-neoplastic agent tazemetostat, has been implicated as an oncogenic driver, with its overexpression or amplification observed in various cancers, including breast, prostate, and bladder cancers. Additionally, mTORC1 has been shown to upregulate EZH2 protein expression, thereby promoting H3K27me3 and controlling cell proliferation [83]. Furthermore, mTORC1 interacts with HMT G9a to suppress autophagy by increasing the repressive histone modification H3K9me2 on promoter sites for genes involved in autophagy [84].

The human Jumonji-C-domain-containing (JMJD) proteins possess both histone lysine demethylase (KDM) and histone arginine demethylase activities [85]. Among these, JMJD1C plays a crucial role in regulating transcription of genes associated with lipid metabolism and synthesis in liver cells. JMJD1C is phosphorylated by mTORC1 at T505 [86]. This PTM enhances recruitment of JMJD1C to the promoters of genes associated with lipogenesis, where demethylation of H3K9me2 enhances the expression of these lipogenic genes. These studies revealed how mTORC1 regulates lipid metabolism through histone modifications.

Histone lysine acetylation stands as a pivotal epigenetic mechanism that profoundly influences chromatin structure and function [87]. Histone acetylation is catalyzed by histone acetyltransferases (HATs). Histone deacetylases (HDACs) counterbalance this process by removing acetyl groups from histones [87]. In yeast, it was observed that TORC1 regulates histone acetylation through the Esa1 HAT complex and Rpd3 HDAC [88]. Interestingly, mTORC1 phosphorylates S4 of the HAT p300, disrupting binding of its catalytic HAT domain to the RING domain [89]. This PTM abolishes intra-molecular inhibition, thereby altering p300 function to suppress autophagy induced by cell starvation while simultaneously stimulating lipogenesis in cells [89]. Such interplay underscores how mTORC1 signaling orchestrates epigenetic modifications to regulate key cellular processes.

Chromatin remodeling complexes play a pivotal role in modulating the accessibility of chromatin to transcriptional and coregulatory machineries by controlling opening of nucleosomes [90]. ARID1A serves as a critical constituent of the canonical BAF (cBAF) complex and functions as a tumor suppressor, one frequently lost through genomic mutations [91]. In hepatocellular carcinoma (HCC), ARID1A is often inactivated by genomic deletion or non-sense mutations. Inactivation of the ARID1A tumor suppressor can also occur post-translationally. Recent work revealed that mTORC1 binds to ARID1A and regulates ubiquitination and proteasomal degradation of ARID1A [92]. The mTORC1–ARID1A axis promotes oncogenic chromatin remodeling and YAP-dependent transcription, thereby facilitating liver cancer cell proliferation in vitro and tumor progression in vivo [92].

DNA methylation is another epigenetic mechanism that regulates chromatin structure and gene expression. It often involves adding methyl groups to the cytosine residues within CpG dinucleotides. This methylation can impact gene expression by influencing DNA accessibility to transcription factors and other regulatory proteins. Promoter hypermethylation often leads to gene silencing, while hypomethylation can increase gene expression [93]. Liver cancer patients with elevated DNA methylation levels and heightened mTOR signaling have the worst prognosis [94]. Subsequent investigations into DNA methylation enzymes revealed that mTOR signaling upregulates both the expression and activity of DNA methyltransferase 1 (DNMT1) [95]. Additionally, mTORC1 enhances the translational efficacy of DNMT1 through a 4E-BP1-dependent mechanism. Notably, concurrent inhibition of mTOR and DNMT synergistically suppresses HCC growth in both in vitro and in vivo models [95]. This study showed that modulating DNA methylation by mTORC1 is involved in hepatocarcinogenesis.

## 6. mTOR Orchestrates the Interplay between Metabolism and Epigenetics

**Figure 4:**
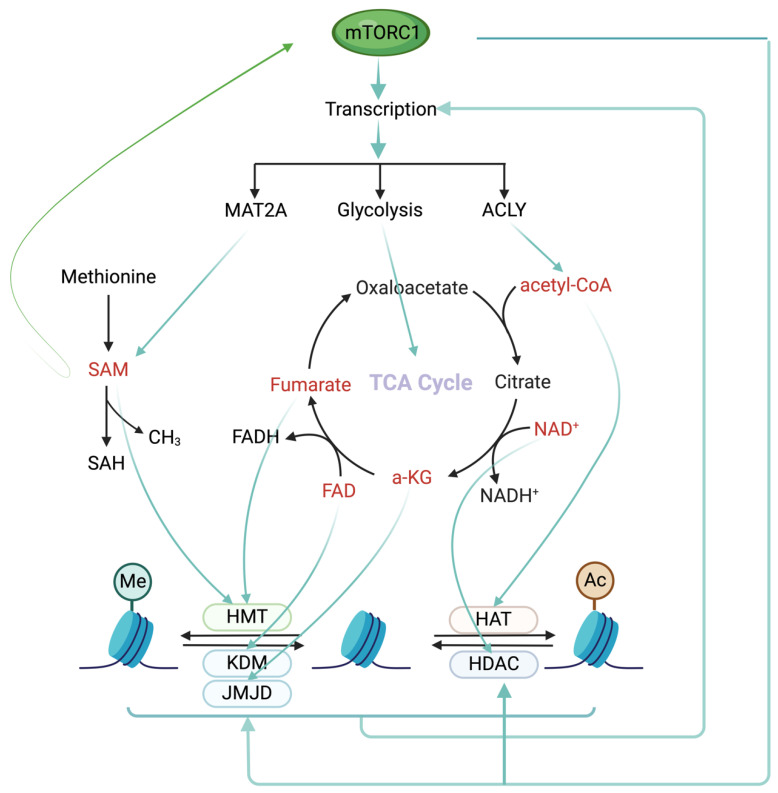
mTORC1 signaling regulates the interplay between cellular metabolism and epigenetics.

As previously discussed, mTOR controls metabolic pathways through epigenetic mechanisms, influencing the level and flux of essential metabolites in both glycolysis and the tricarboxylic acid (TCA) cycle. For example, SAM synthesis from methionine and ATP is regulated by the mTORC1–Myc–MAT2A axis [96]. mTORC1 stimulates ACLY expression, facilitating synthesis of acetyl-CoA through the mTORC1–SREBP signaling axis [97]. Many metabolites, including acetyl-CoA, flavin adenine dinucleotide (FAD), fumarate, α-ketoglutarate (α-KG), nicotinamide adenine dinucleotide (NAD^+^), nicotinamide (NAM), and S-adenosylmethionine (SAM), can also impact histone and DNA modifications by serving as either cofactors or substrates for epigenetic enzymes [98,99]. These metabolites, through influencing the activity of mTORC1 signaling or epigenetic enzymes, can serve as feedback mechanisms to finetune the expression programs in response to metabolic demands (Figure 4).

S-adenosylmethionine (SAM) plays a crucial role in histone methylation, a key epigenetic modification governing gene expression. SAM serves as a methyl group donor for histone methyltransferases, enzymes that catalyze the addition of methyl groups to specific lysine or arginine residues on histone proteins [100], as well as a methyl group donor for DNA methytransferases [94]. This methylation can either activate or repress gene transcription, depending on the specific histone residue targeted and the extent of methylation, by influencing chromatin structure and accessibility. It is intriguing to note that SAMTOR, an SAM-binding protein, serves as a sensor for SAM, exerting a negative regulatory effect on mTORC1 [101]. When SAM levels are low, SAMTOR interacts with GATOR1, leading to the suppression of mTORC1 activity. Conversely, the binding of SAM to SAMTOR disrupts its interaction with GATOR1, resulting in mTORC1 activation. This discovery unveils a feedback regulatory mechanism linking one-carbon metabolism with its upstream regulator, offering precise modulation of the interplay among mTORC1 signaling, one-carbon metabolism, and the epigenetic control of gene expression.

Histone demethylation is an equally important counter process in epigenetic regulation, in which flavin adenine dinucleotide (FAD) plays a significant role. Enzymes, such as KDM1A and KDM1B, facilitate histone demethylation through an FAD-dependent amine oxidation reaction [102]. FAD serves as an electron carrier, facilitating transfer of electrons during demethylation. α-KG functions as a co-substrate for enzymes involved in demethylation of histones and DNA [103]. Specifically, α-KG-dependent dioxygenases, including the Jumonji C (JmjC)-domain-containing histone demethylases (e.g., KDM4 and KDM6), utilize α-KG as a co-substrate to catalyze methyl group removal from histone lysine residues and DNA cytosine nucleotides, respectively [102]. Availability of SAM, FAD, and α-KG is essential for proper functioning of these histone methyltransferases and demethylases. Additionally, DNMT1 and DNMT3A were reported to be major methyltransferases that consume the excess SAM to change DNA methylation status in a lung cancer model [104]. These findings highlight the interplay between cellular metabolism and epigenetic regulation.

While the level of acetyl-CoA is regulated by mTORC1, acetyl-CoA itself also serves as a co-substrate for histone acetylation. HATs utilize acetyl-CoA as the acetyl donor for histone acetylation reactions. ATP citrate lyase (ACLY) converts citrate into acetyl-CoA, which provides the acetyl groups necessary for histone acetylation [105]. Integrated analyses demonstrated that mTORC1 increased the protein expression of EZH2, while mTORC2 controlled the production of SAM in human glioblastoma (GBM). This resulted in collaborative regulation of EZH2’s methytransferase activity and H3K27 hypermethylation, subsequently enhancing tumor cell survival both in vitro and in vivo [106]. This finding provides an example of the cooperativity of mTORC1 and mTORC2 in promoting tumor progression through interplay between epigenetics and metabolism. Disruptions in these interplays can have implications in human diseases, particularly cancer.

## 7. Future Perspectives

mTOR signaling plays crucial roles in cellular growth and metabolism by controlling gene expression programs through various molecular mechanisms, including direct modulation of transcription factors, epigenetic enzymes, and chromatin remodelers. mTOR directly influences epigenetic/chromatin regulation and mediates the intricate interplay between metabolism and epigenetic processes. The regulatory pathway of mTOR in transcriptional machinery and epigenetics is not a simple linear progression. Instead, it is a complex network, akin to a vast and intricate roadmap with multiple interconnected routes and feedback loops.

Despite recent strides in understanding nuclear mTOR signaling, our grasp of the intricate mechanisms by which mTOR influences nuclear processes remains limited. Several key questions persist. Firstly, while cytoplasmic and nuclear mTORC1 are largely regulated by the same upstream factors, the spatial and temporal organization of the nuclear mTORC1 signaling pathway in response to nutrient stimuli requires further elucidation. Secondly, although there is some indication of mTORC1’s non-catalytic involvement in transcriptional control, a comprehensive mechanism has yet to emerge. Thirdly, the reasons behind mTORC1 kinase’s binding to specific chromatin regions, such as enhancers and promoters, remain unclear. One hypothesis posits that mTORC1 may serve as a scaffold, facilitating the proximity of transcription factors, epigenetic enzymes, and chromatin remodelers to enhance the co-regulation of these substrates via phosphorylation. This scaffolding function could lead to more robust responses to both extracellular and intracellular cues.

Finally, the role of nuclear mTORC1 signaling in the context of human diseases such as cancer, remains to be better defined. Considerable efforts will be needed to deconvolute detailed molecular mechanisms. Activation of mTOR promotes tumorigenesis and metastasis. mTOR inhibitors, such as rapamycin analogs like everolimus and temsirolimus, are anti-cancer drugs, approved by US FDA for various indications. However, the clinical efficacy of rapalogs remains limited due to the broad role of mTORC1 in normal physiology of many vital organs and lack of reliable predictive biomarkers. In this regard, targeting specific mTORC1-specific nuclear processes may offer better efficacy/toxicity profiles. Enhanced understanding of how nuclear mTOR orchestrates growth/metabolic reprogramming and transcription within the cancer microenvironment will facilitate discovery and development of more selective therapeutic strategies.

## Data Availability

Not applicable.

## Abbreviations

mTOR, mechanistic target of rapamycin; PI3K, phosphatidylinositol-3-kinase; mTORC1/2, mTOR complex 1/2; rDNA, ribosomal DNA; Raptor, regulatory-associated protein of mTOR; mLST8, mammalian lethal with Sec13 protein 8; PRAS40, proline-rich AKT1 substrate 1; DEPTOR, DEP-domain-containing mTOR-interacting protein; S6K1, S6 kinase 1; 4E-BP1, eukaryotic initiation factor 4E-binding protein; SREBP1, sterol regulatory element-binding protein 1; rRNA, ribosomal RNA; tRNA, transfer RNA; RICTOR, rapamycin insensitive companion of mTOR; mSIN1, stress-activated map kinase-interacting protein 1; Protor, regulatory component proteins associated with RICTOR; Akt, protein kinase B; PKC, protein kinase C; SGK1, serum/glucocorticoid regulated kinase 1; ChREBP, carbohydrate response element-binding protein; FoxO, Forkhead box O; TFEB. transcription factor EB; PTM, covalent post-translational modification; HMT, histone methyltransferase; EZH2, Enhancer of Zester 2; PRC2, Polycomb repressive complex 2; H3K27me2 and -me3, di- and tri-methylation of H3K27; SAM, S-adenosylmethionine; MAT2A, methionine adenosyl transferase 2 alpha; JMJD, Jumonji-C-domain-containing; KDM, histone lysine demethylases; HATs, histone acetyltransferases; HDACs, histone deacetylases; ACLY, ATP citrate lyase; GCN5, general control non-repressed protein 5; TFEB, transcription factor EB; PGK1, phosphoglycerate kinase 1; SWI/SNF, switch/sucrose-non-fermenting; FAD, flavin adenine dinucleotide; α-KG, α-ketoglutarate; NAD^+^, nicotinamide adenine dinucleotide; NAM, nicotinamide; rapalog, rapamycin analog.
